# Bodily Self-Disturbances and Hallucinations in Schizophrenia

**DOI:** 10.1093/schbul/sbaf063

**Published:** 2025-10-06

**Authors:** Anne Giersch, Francesca Ferri, Sohee Park, Judy L Thompson, Clara S Humpston

**Affiliations:** University of Strasbourg, INSERM U1329 Strasbourg Translational nEeuroscience and Psychiatry (STEP) team Psychiatry, University Hospital of Strasbourg, ITI Neurostra, France; Department of Neuroscience, Imaging and Clinical Sciences (DNISC), “G. D’Annunzio” University of Chieti-Pescara, 66100 Chieti, Italy; Institute for Advanced Biomedical Technologies, “G. d’Annunzio” University of Chieti-Pescara, 66100 Chieti, Italy; Department of Psychology, Vanderbilt University, Nashville, TN 37240, United States; Departments of Psychiatry and Neuroscience, University of Rochester Medical Center, Rochester, NY 14642, United States; Department of Psychology, University of York, York, YO10 5DD, United Kingdom; School of Psychology, University of Birmingham, Birmingham, B15 2TT, United Kingdom

**Keywords:** self-disturbances, hallucinations, schizophrenia, interoception, timeperception, peripersonal space, vestibular system

## Abstract

**Background and Hypothesis:**

Auditory verbal hallucinations experienced by people with schizophrenia are primarily described in terms of unusual perceptual experiences. However, they also involve profound self-disturbances. More specifically, they are deeply intertwined with disorders of the sense of bodily self. Sometimes hallucinations in schizophrenia are experienced as invading the body, whereas other times they seem to help to delineate a frontier between oneself and others.

**Study Design:**

As one of the working groups at the 2023 biennial meeting of the International Consortium on Hallucination Research (ICHR), here we review the disorders of the sense of bodily self in schizophrenia and delineate the link between these disturbances and auditory verbal hallucinations.

**Study Results:**

We report findings suggesting that peripersonal space, interoception, timing, and the vestibular system may all be disturbed in schizophrenia and that there may be reciprocal influences between bodily self-disturbances and hallucinations in this condition.

**Conclusions:**

Schizophrenia is associated with multiple sensory disturbances that contribute to the sense of bodily self. How these disturbances interact remains to be explored. The relationship between weakened sense of the bodily self and hallucinations in schizophrenia is rather complex, with some evidence suggesting that hallucinations in schizophrenia may both contribute to disturbances in the sense of self and, in some cases, temporarily restore self-other boundary. Future studies are needed to establish the causal and bidirectional aspects of these relationships. We can envisage several therapeutic approaches based on the available findings, which will likely have to be adapted to the patients’ own needs.

## Introduction

Hallucinations are common in schizophrenia (SZ), occurring in approximately 70%-80% of individuals with this condition.^1,2^ Although hallucinations in SZ can occur in any sensory modality, auditory hallucinations, most notably auditory *verbal* hallucinations (AVH)—or voices—are the predominant type experienced.^2^

The *Diagnostic and Statistical Manual of Mental Disorders* (5th ed; DSM-5)^3^ defines hallucinations as clear perceptual experiences that occur in the absence of corresponding external stimuli. It is notable that this standard definition of hallucinations does not reference what seems to be a core feature of how they are experienced in psychosis, namely self-disturbances.^4–7^ Indeed, the exclusive focus on the perceptual aspects of hallucinations, along with an emphasis on unimodal sensory experiences, belie the complexity, heterogeneity, and nuances of hallucinations in psychosis.^4–8^ It is our aim here to delineate the link between AVH and the sense of bodily self and to describe how there may be reciprocal influences between bodily self-disturbances and hallucinations, in SZ.

Multimodal hallucinations present fascinating challenges and opportunities for the investigation of the bodily self. They are defined as hallucinations that co-occur in different sensory modalities, which are related in thematic content and occur simultaneously or serially.^9^ This definition highlights the fact that despite an occurrence in different sensory modalities, the sensory perceptions associated with the hallucinations are not necessarily integrated within one unique, “multisensory” perception. In the context of SZ and related psychoses, multimodal hallucinations can manifest not only as distinct perceptions but can also be experienced as something subtle and ineffable that influences the entire body.

Several recent phenomenological investigations have made clear that the experience of AVH often includes somatic aspects, such as accompanying bodily sensations (eg, tingling, heat, pressure, or tension^10–12^), and for some, the sense of feeling their voices in parts of their body.^4^ Co-occurring dissociative experiences, such as feeling disconnected from one’s body or a reduced sense of body ownership, have also been reported.^10,12^ Such findings illustrate how the focus on unimodal perceptual experiences in both research and clinical settings likely impedes fully understanding the phenomenology and related mechanisms of AVH and other hallucinations in psychosis. This point is underscored by the results of a small but innovative study that employed multimodal unusual sensory experience (MUSE) maps to prospectively investigate the location and nature of bodily feelings associated with hallucinations, specifically among young people who were receiving early intervention services for psychosis.^13^ All the participants reported that their hallucinations were accompanied by bodily sensations in some form, with the specific location and nature of the feelings varying across participants. Several participants indicated that these hallucination-related sensations extended into their peripersonal space, as notably illustrated by Melvin et al.^13^ Other studies employing similar methods, such as emotional embodiment body mapping tasks,^14–16^ also support the notion that multimodal hallucinations and anomalous bodily feelings are highly related, perhaps almost indistinguishable for some.

If hallucinations and body sensations are associated, they may also sometimes interact. Interestingly, such relationships can differ across individuals. In the following sections, we describe the case in which hallucinations seem to invade and thus weaken the sense of self, and the case when they may paradoxically bolster the sense of self.

## Reciprocal Impacts of Bodily Self-Disturbances and Hallucinations

### When Hallucinations Invade the Self

Non-clinical voice hearers (NCVH) recruited from spiritualist or psychic communities often report experiencing some level of control over their voice-hearing experiences, including the onset and offset of their voices. In addition, they often describe seeking out their voices, which are likely to convey positive content and result in feelings of comfort and other positive emotions.^8,17,18^ In contrast, many (although not all^19^) individuals with psychosis report having little, if any, control over their AVH.^4,8^ They are also significantly more likely to hear voices that contain abusive or negative content and experience negative emotional responses to their voices, such as distress.^8,18^ Gold and colleagues^18^ observed that SZ participants with AVH endorsed significantly more Schneiderian first-rank symptoms (FRS), including delusions of passivity/control, compared to both NCVH and SZ participants without AVH. These observations cohere with the phenomenological literature, which indicates that individuals with psychosis often experience their AVH as intrusive yet compelling, and thus difficult to ignore. Some describe the sense of being invaded by voices that they cannot control without any way to escape.^5,11^ Many individuals with AVH experience related passivity phenomena,^18^ which may lead to the development of delusions of passivity/control, such as believing that the voices, other forces, or people have direct access to their thoughts, and/or that the voices can control or influence their thoughts, emotions, and actions.^20^ These features also characterize thought-like voices. Many individuals who report AVH indicate that at least some of their voices lack the qualities of a true perception and instead are either more thought-like or are somewhere in between full perceptual experiences and thoughts.^4,10^

Such soundless voices lack the sense of “mineness” that characterizes typical thoughts. Instead, they are experienced as coming from an external source, often as thoughts being transmitted or inserted into one’s mind by an outside entity.^21^ It has been noted that in many cases, these passivity phenomena seem to be inextricably linked with AVH in SZ.^4,6,7^ What is striking is the immediacy and physicality of such feelings and experiences. For example, when discussing thought insertion and related phenomena, perhaps not many clinicians would consider them as hallucinations but as delusions of thought interference. The thoughts involved are not “heard” by the patient and thus cannot be considered AVH according to the standard definition of hallucinations. However, phenomenological research indicates that for many patients, thought interference is more akin to, if not identical, to somatic hallucinations involving the entire body. In other words, experiences of thought interference can go far beyond a mere belief about the source of one’s thoughts. Rather, it is inexplicably perceived as an attack on or a breach into, one’s innermost sense of self. The impact of these experiences can lead to heightened feelings of threat, dread, and existential annihilation, which may sometimes contribute further to anomalous bodily sensations, thus forming a vicious circle.

These passivity experiences can lead to profound changes in beliefs regarding the nature of reality. However, at their core, they reflect disturbances in the basic sense of self, including a loss of agency, that is, a diminished sense of “mineness” and control of one’s thoughts and bodily self, and disruptions to the boundaries of the self.^22,23^ Raballo wrote of this latter disturbance, “…the whole interiority of the person is exposed, her thoughts and desires become somehow accessible and transparent to others and the immanent privacy of her embodied psychic stream is lost” (p. 140).^24^ Parnas et al.^6,25^ have emphasized that AVH can be experienced as “hyperproximate,” which alters the individual’s sense of their innermost self: “…the patient feels totally exposed, without any possibility of evasion or retreat, because the patient cannot escape from himself…” (p. 85).^6^ It is thought that this disrupted permeability of the self and related alterations in agency may represent a central disturbance in psychosis.^4,6^

Of note, and as further elaborated by some of us,^26^ it is possible that these experiences of self-disturbance in psychosis begin with a perplexity regarding external or commonsensical reality. These are all important considerations when studying the complex interactions between self and external reality, and how they relate to one’s experience of the body.

### When Hallucinations May Strengthen the Sense of Self

Contrary to the case where hallucinations seem to invade the self, multimodal hallucinations can manifest outwards from one’s subjectivity as fully formed and personified external agents. Instead of experiencing intrusions into their selfhood by these entities, patients might experience personified hallucinations as autonomous beings with whom they can communicate. There is now extensive research focusing on sensed/felt presence in individuals with and without psychiatric diagnoses (see Barnby et al.^27^ for a comprehensive overview). Curiously, the experience of sensing such a presence can in some cases help to demarcate the boundary between self and other, therefore promoting a stronger ego-boundary and potentially re-establishing an intact sense of self (see also the discussion on the peripersonal space,^28^ below).

Thus, some hallucinations involving the bodily self may paradoxically bolster the sense of integrity of the body, which means that such disturbances are not necessarily always maladaptive. Recent phenomenological accounts by individuals with SZ suggest that their delusions and hallucinations are often more real than consensus reality,^29,30^ which may in turn help them regain a sense of realness in their bodily self, at least temporarily. It may, for some, be a case of reintegration following dissolution, reclaiming one’s self by relinquishing control over the same self, and returning to feelings of intersubjective situatedness. We have previously proposed that certain psychotic symptoms and self-disturbances could act as an unreliable yet desperately needed “adhesive” for the integrity of self.^31^ For example, as hallucinations often possess a distinct quality or realness compared with their own diminished sense of self, they may reassure some patients about the existence of the bodily self and allow a reassertion of a sense of coherence, even if an erroneous or unreal one. Certainly, such a stance may not be adaptive in the long run, but they may nevertheless lessen the initial emotional impact and threat posed by persistent and complex hallucinatory experiences on the lived body. Nonetheless, we must acknowledge phenomenological findings^32^ suggesting that hyper-reality can also be expressive of a diminished sense of self and involve a disruption of intersubjective situatedness. The paradoxical coexistence of hyper- versus hypo-real experiences in SZ represents an urgent research need.

## Self-disturbances and Disorders of the Sense of Bodily Self in Schizophrenia

Given the close link between hallucinations and disorders of the bodily self, it is important to investigate bodily self-disturbances to better comprehend hallucinations in psychosis. At least 2 levels of selfhood have been described (although the levels of selfhood are conceptually diverse and are subjects of ongoing debate): the minimal self and the narrative self. The *minimal self*, also known as *ipseity,* is the basic, foundational level of selfhood^33^ through which one acquires the first-person perspective. Importantly, ipseity is thought to be pre-reflexive such that the experience of the minimal self is implicit and automatic. In contrast, the narrative self is constructed from binding episodic memories of personal history, learning, and social experiences across time, resulting in explicit self-identity and a coherent self-story incorporating our past and used to build our future.^34^ In the case of SZ, both minimal self and narrative self are altered. However, we focus on the minimal self in the following sections, given that it is tightly linked with the sense of bodily self.

An implicit awareness of one’s body as a unified entity with fixed and solid boundaries allows us to distinguish the self from the environment. This experienced unity of self and body is necessary for adaptive social interactions, but this core sense of the bodily self is disrupted in SZ throughout the course of illness.^35–40^ Previous evidence suggests that around 70% of patients with SZ report bodily self-related abnormalities,^41^ ie, disturbances of the basic sense of self.^39,42,43^ First-person accounts of SZ are replete with descriptions of highly salient and aberrant self-experiences such as the loss of a coherent sense of self, blurring between the self and other, loss of body ownership and agency, and even out-of-body experiences.^37,43,44^ The phenomenology of losing one’s own self-boundary is beautifully captured by Elyn Saks in her memoir.^45^ For example, she writes, *“I am dissolving…like a sandcastle with all the sand sliding away.”*

Interestingly, what may be specific to the SZ phenotype are the disembodiment (ongoing bodily feelings of disintegration/violation) and objectification/mechanization (one’s body experienced as an object-like mechanism) of the lived body. For example, such alterations do not map neatly onto the bodily experiences of individuals with affective disturbances (eg, severe depression), who are more likely to experience mood-congruent abnormal bodily sensations, such as reduced sensitivity towards interoceptive signals, or the feeling of emptiness/absence, or a lack of liveliness. Individuals with SZ may report vivid experiences of inserted thoughts or external forces entering a specific part of their body, or that their organs have been replaced or even moved outside. It must be noted that these experiences are sometimes intrinsically and distinctively perceived by the patient as *actual* sensations and not simply delusions. The specificity of these bodily experiences to SZ are the subject of ongoing debate; for example, seemingly similar phenomena can also be found in depersonalization-derealization disorders.^46^ Nevertheless, in SZ, the “as if” feeling towards bodily disintegration and objectification is usually absent; rather, unlike in depersonalization, aberrant bodily experiences frequently lead to delusional elaborations or confirmations of the reality of hallucinations.

Accumulating evidence indicates that such anomalous self-experiences are present long before the onset of SZ and that they may lead to social impairments and disconnection^39^ and likely impede full recovery.^47^ Indeed, self-disturbance was central to Bleuler’s conceptualization of SZ.^48^ Thus, understanding the specific disturbances in the sense of bodily self in SZ and characterizing their underlying neurocognitive mechanisms could lead to the identification of novel treatment targets to improve functional outcomes. We will first examine the concept of the sense of self in neurotypical individuals.

## The Neurocognitive Mechanisms Underlying Self-Disturbances in Schizophrenia

### The Sense of Bodily Self in Neurotypical Individuals

The nature and definition of the self have been debated for centuries (at least since Descartes) and continue to be debated across multiple disciplines.^49,50^ Several authors have suggested that during infancy, our sense of the bodily self develops through repeated dynamic multisensory interactions with the environment.^51,52^ Thus, a stable and clearly defined self-boundary is shaped by our interactions with the social world and, in turn, provides us with a framework to interpret and integrate our somatic signals. In neurotypical individuals, this results in an effortless self-other distinction that forms the basis of the minimal self.^53^

The fact that we have one unique body seems self-evident. Yet, the sense of the body includes many aspects, i.e., boundary, body location, body ownership, agency, and body continuity in time. It rests on the multisensory integration of exteroceptive, proprioceptive, and interoceptive signals. However, what “multisensory integration” means in this case may require clarification. Full integration can occur for audio-visual signals, leading to a new unified percept. For example, in the McGurk illusion,^54^ simultaneously presented auditory and visual information in the form of an audio clip of a spoken syllable (“BA”) paired with a silenced video of a speaker saying the “GA” leads to a new auditory percept entirely, that is, “DA.” This kind of integration impedes us from detecting the original visual “GA” and auditory “BA.” It is a type of fusion that differs from the integration underlying the sense of self. As a matter of fact, despite one unique sense of self, we do distinguish between the bodily sources of information and do not fuse them into a unique sensory percept. This paradox of one body and multiple information sources has led to several models that go beyond the scope of the present manuscript.^55,56^

A wide range of sensory information feeds into the sense of self. The sense of bodily self is thought to arise from the interaction between multiple bottom-up sensory signals and top-down processes of spatiotemporal integration.^57^ Similarly, proprioception and monitoring of actions support the development of agency (the sense of control over one’s actions). For example, when we reach for our coffee mug and grab it (exercising agency), we get sensory feedback resulting from our actions. Such reaching cycles repeated thousands of times scaffold the generation of a mental representation of one’s own body.^51,52^ The mental representation of the body yields a sense of ownership, agency, and self-location, as well as a sense of self-other distinction, leading to “the special perceptual status” of one’s own body, which makes bodily sensations seem unique to oneself.”^33,58^ Thus, the agency supports the development of the sense of body ownership but once a stable sense of body ownership is established, it becomes independent of agency *most of the time* (e.g., my arm is mine even when someone else moves it).

In sum, a variety of sensory inputs contributes to the sense of self. It is thus possible to deconstruct the basic notion of ipseity itself into components that can be experimentally manipulated and quantified, which will help to elucidate the mechanisms underlying the sense of bodily self and their alterations in conditions like SZ.

### The Sense of Bodily Self in Individuals with Schizophrenia

It has been shown that body ownership, agency, embodiment, and temporal integration are disrupted in SZ.^15,37,59,60^ There is a vast literature on agency disorders in schizophrenia^61–63^ and on multisensory integration.^64–66^ Bodily self-illusions, such as the rubber hand illusion (RHI), have been used to probe these functions in SZ. During this illusion, a rubber hand is positioned next to the real hand, which is hidden from view. Both hands are brushed simultaneously, leading the participant to see the brush strokes on the rubber hand while feeling the tactile stimulation on the real hand at the same time. After a while, the synchronous visual and tactile stimulation leads the participant to endorse the rubber hand as theirs. The role of visuo-tactile integration in this illusion suggests that multisensory integration is a mechanism supporting the sense of self. It has been shown that the RHI is enhanced in patients with SZ, suggesting that their body boundaries and ownership are more fragile.^37^ More recent results suggest additional difficulties, however. In the following sections, we review how several additional aspects of the bodily self that have received less attention may be affected in SZ.

### The Peripersonal Space and Self-Other Boundary

Disrupted self-other processing may be framed as the manifestation of an abnormal representation of the body in relation to the regions of space that mark the perceptual border between the self and others. The space surrounding the body where the self and objects can physically interact is known as the peripersonal space (PPS).^67–70^ To achieve the everyday miracle of moving through the world without colliding with objects or people, the brain continuously integrates multisensory exteroceptive and proprioceptive signals to predict the self-location in relation to the environment and maintain this sense of PPS, thus protecting the body from potential danger. The integrity of this buffer zone between the self and others is closely related to our capacity for self-other distinction.

Given the reports of blurred self-boundary and difficulties with self-other distinction in SZ, one would expect SZ to be associated with PPS abnormalities. Surprisingly, an auditory-tactile integration study using dynamic approaching sounds and tactile vibration found that in individuals with SZ, PPS size was contracted and had a sharper boundary compared to that of controls.^71^ Hence, in the auditory space, the self-other boundary seems more clearly defined in SZ, which is puzzling, given the multitude of bodily self-disturbances that characterize this condition. As discussed above, it is possible that other features of SZ, such as hallucinations, may serve to actually counter aspects of these self-disturbances. Lee et al.^28^ indeed found an intriguing association between *sharper* self-boundary and severity of hallucinations. Specifically, sharper (clearer) self-other boundary was observed in individuals with SZ with more severe hallucinations, suggesting the possibility that the hallucinatory “other” may be helping to define one’s boundary and thereby restoring the spatial aspect of the self. This idea clearly requires further investigation, given classic phenomenological studies (eg, Straus, Naudin, Azorin, and others)^72^ indicating that hallucinatory others often have a particularly elusive status.

### Interoception

Beyond body ownership and agency, Seth^73^ has proposed an interoceptive predictive coding model of selfhood and consciousness. Interoception refers to the perception of the internal state of the body.^73^ Interoception plays a crucial role in cognitive, behavioral, emotional, and social functioning through homeostasis and allostatic responses.^74^ Beyond its role in basic survival (eg, hunger signals prompt us to eat), interoception is also central to the sense of self,^75^ and influences emotional states and social functioning.^76^ The steadiness of implicit interoceptive signals provides an anchor to our physical self-experiences, and is thought to be the source of the unitary sense of self.^77^ In this context, Seth has proposed that embodied presence, i.e., the sense of inhabiting one’s body, and sense of agency arise from the successful integration of interoceptive predictions (sense of what happens next), autonomic signals, and bodily response signals.

Empirical evidence suggests that interoception and sense of bodily self are related in a bidirectional manner. For example, looking at one’s own face has been shown to increase interoceptive accuracy, i.e., one’s ability to perceive internal bodily signals.^78^ Importantly, interoceptive ability predicts the perceived size of one’s PPS (ie, the space of the bodily self). Ardizzi & Ferri^79^ have shown an association between high interoceptive accuracy and smaller PPS. Interoception is also linked to the sense of body ownership. The rubber hand illusion (RHI)^80^ is enhanced in individuals with lower interoceptive accuracy.^81^ Likewise, those with low interoceptive accuracy are more likely to experience the enfacement illusion (which is akin to the rubber hand illusion, but applied to the face).^82^ These findings suggest that those with better interoceptive ability might have a stronger sense of the bodily self, given that they are less susceptible to these bodily self-illusions. The fact that individuals with SZ are more susceptible to such illusions leads to the question of their interoceptive ability. Indeed, it has been consistently observed that patients with SZ have difficulties in feeling their own heartbeats, indicating a deficit in interoceptive accuracy.^83–85^ Furthermore, it has been shown that such impairments are not explained by alterations in exteroceptive perception.^86^ These results certainly suggest that this aspect of the bodily self deserves to be explored further.

### Timing

We intuitively think that our perception faithfully reflects the outer world, that it is as continuous as time seems to be, and that we ourselves are continuously in contact with our environment. Patients with SZ report a fragmentation of thought and experience of time,^87–89^ with first-hand accounts suggesting alterations in both the sense of time continuity and the continuity of oneself in time: “Time splits up and doesn’t run forward anymore. There arise uncountable disparate now, now, now, all crazy and without rule or order. It is the same with myself. From moment to moment, various ‘selves’ arise and disappear entirely at random. There is no connection between my present ego and the one before” (Kimura, cited in Fuchs^87^). Such reports have led phenomenologists to hypothesize a link between the sense of self and timing.^87,89,90^ Timing has also been explored experimentally. The roots of time fragmentation seem to be best found in what allows time to have a structure, that is, succession, order, and simultaneity.^91^ This ability to structure events in time seems to be altered in patients with SZ^64,92–95^ (ie, patients with SZ need more time between successive events than neurotypical individuals to detect that the events are asynchronous).

Paradoxically, however, patients with SZ are more affected than neurotypical individuals by short, subconscious asynchronies. Interestingly, this is also the case for actions. These alterations have been shown to be related to the sense of self. When patients with SZ perform a voluntary pointing action, they react abnormally to undetectable and unpredictable delays in the sensory feedback.^96^ Moreover, those abnormal reactions affect the patients’ feeling of control: when there is a delay in the sensory consequence of the action, patients with SZ feel like they do not control the action. It has been proposed that abnormal temporal prediction impedes patients from discarding subliminal delays. Those delays are not conscious but would disrupt the passage of time and its continuity, thus affecting the sense of self. Two studies have confirmed that patients with SZ who are experiencing disorders of the sense of self^97^ (as evaluated with a phenomenological scale) do not benefit from the passage of time when preparing for a target.^98,99^ These studies reinforce the idea of a link between timing and self-disturbances.

### The Vestibular System

The sense of balance, maintained by the vestibular system, is the only sensory modality to our knowledge that, when disturbed, leads to disorders of the bodily sense of self that are similar to those observed in psychiatric disorders.^100^ Patients with a vestibular syndrome indeed experience derealization, depersonalization, and out-of-body experiences. Whether this observation is or is not relevant for SZ remains to be investigated. Crucially, the vestibular system provides information about the body and head displacements in real time, whether displacements are voluntary or passive. It is thus no surprise that impaired transmission of vestibular information leads to disorders of the sense of self. However, evidence for vestibular impairments in SZ is even more scarce than for interoception. A difficulty here is to make a distinction between vestibular and cerebellar impairments.^101^ Nonetheless, several authors have suggested vestibular impairments in SZ, based on observations of increased sway, altered posture and balance,^101^ or abnormal motion perception.^102^ Those alterations are independent of antipsychotic intake.^103^ Visual over vestibular dominance does not seem altered in patients, however,^104,105^ although this may change with chronicity.^104^

## Perspectives and Future Directions

### The Need to Study the Body in its Full Complexity

Bodily self is a multifaceted construct that is shaped by multiple sensory and sensorimotor signals from the body and the environment. Self-disturbances, which are core components of SZ, may involve several aspects of the bodily self, such as the peripersonal space representation,^28,71,106,107^ the body structural representation,^108–110^ the sense of body ownership and agency,^37,108,111–114^ interoceptive accuracy and sensitivity,^79,85,86^ and the sense of self-continuity in time.^60^ Psychosis proneness and SZ are also associated with alterations at the level of multiple sensory and sensorimotor mechanisms underlying the different facets of the bodily self.^18,64,66,95^

A limitation of this work is that alterations have mostly been investigated separately in SZ and subthreshold psychosis. Consequently, we do not know which alterations are primary and whether different abnormalities may be compensatory. Simultaneous investigation of diverse sensory, sensorimotor, and bodily-self dimensions could be revealing and useful, especially for the link with hallucinations.

Associations between impairments in the bodily self and clinical symptoms, including but also beyond hallucinations, also need to be further clarified. As it stands, the temporal aspects of multisensory integration are associated with positive symptoms in SZ and to psychotic-like traits in individuals with high schizotypy.^64,95,115–117^ The paradoxical sensitivity to short delays has been related to positive symptoms using a motor task,^99^ whereas disorders in consciously detecting asynchronies are correlated with disorganization,^118^ and time prediction at the level of seconds is linked with the disorders of the minimal self.^98,99^ Conversely, abnormal extension of peripersonal space boundaries is mostly associated with negative symptoms.^71,119–122^ Systematic investigations into the potentially specific contributions of various sensory and body representation deficits to the positive, negative, and disorganized dimensions of SZ are needed to clarify whether certain deficits alone or in combination contribute to specific categories of symptoms.

To understand bodily self-disturbances in SZ, which requires the study of the body in its full complexity, research should extend to the less explored sensory dimensions of body perception and awareness, like interoception and the vestibular system. Most importantly, we need to understand how the different dimensions interact to generate a sense of unitary and continuous body in time, that is, the brain’s ability to sense, interpret, and integrate signals originating from within the body at both conscious and unconscious levels.

The temporoparietal junction (TPJ) appears to play a key role in the late integration of bodily-related information,^123^ and thus further study of this brain region, including its link to various symptoms in SZ, should advance our understanding of the interface between bodily self-disturbances and hallucinations. The TPJ is central to implicit body processing, such as maintaining an accurate map of body location, perception of self-body unity, and sense of body ownership.^45^ Several studies have already reported decreased activation of the TPJ in SZ during a range of tasks involving some aspect of the bodily self,^124^ for example, ownership,^125^ agency,^126^ or retrieval of self-referential information in memory.^127^ Decreased connectivity in a network including the TPJ has also been observed in SZ during agency tasks.^128,129^ Future studies will have to determine if what is seen at the level of the TPJ is causal in driving alterations of the bodily sense of self in SZ, or whether it is the consequence of impairments at an earlier level.

Beyond the conscious level, patients with SZ seem to exhibit abnormalities at the unconscious level of brain–body connections. For example, they display aberrant heartbeat-evoked potentials (HEP), reflecting cortical processing of cardiac signals,^84^ as well as altered causal coupling pathways within the central-autonomic network, including abnormal cardiac and respiratory influences on central activity.^130^ As noted above, timing impairments are also observed at the subconscious level.^96^ The relative role of early sensory processing and late integration of bodily-related information will have to be determined.

### Potential for Movement- or Body-Based Therapies?

Given the potential contributions of SZ-related self-disturbances to how AVH are typically experienced in psychosis, namely as uncontrollable, invasive, and threatening, along with the distress and social impairments associated with anomalous self-experiences, body-centered rehabilitation interventions have the potential to meet a host of unmet therapeutic needs in SZ. This perspective is supported by evidence such as the observed expansion of peripersonal space boundaries following sensory-motor training in individuals with SZ.^131^ Additionally, patients with SZ may benefit from existing perceptual training programs designed to enhance multisensory temporal processing.^132^ Impairments in interoceptive accuracy could also offer a novel therapeutic target. For instance, mindfulness practices and specialized training or treatments aimed at improving interoceptive awareness may hold potential for addressing these abnormalities.^133,134^ Finally, therapeutic interventions utilizing virtual reality have been proposed to enhance bodily self-consciousness through synchronized multisensory stimulation (visual-tactile), interoceptive feedback, and the expansion of peripersonal space (visual-tactile and audio-tactile).^135^

## Conclusions

We reviewed the literature to elucidate the relationship between 2 core features of schizophrenia: bodily self-disturbance and hallucinations. Although many of the published studies of hallucinations in schizophrenia have focused on AVH, it is important to acknowledge the multisensory nature of perception, hallucination, and the multiple mechanisms involved in our own sense of the bodily self. The relationship between weakened sense of the bodily self and hallucinations in schizophrenia is rather complex, with some evidence suggesting a role of hallucinations in temporarily restoring self-boundary. Future studies are needed to establish the causal and bidirectional aspects of these relationships. Lastly, given that self-disturbance may contribute to the particularly devastating aspects of AVH in psychosis, as well to poor social outcome in schizophrenia, it is imperative that therapeutics should target aberrant self-experiences.
